# Metabolic marker-assisted genomic prediction improves hybrid breeding

**DOI:** 10.1016/j.xplc.2024.101199

**Published:** 2024-11-29

**Authors:** Yang Xu, Wenyan Yang, Jie Qiu, Kai Zhou, Guangning Yu, Yuxiang Zhang, Xin Wang, Yuxin Jiao, Xinyi Wang, Shujun Hu, Xuecai Zhang, Pengcheng Li, Yue Lu, Rujia Chen, Tianyun Tao, Zefeng Yang, Yunbi Xu, Chenwu Xu

**Affiliations:** 1 Key Laboratory of Plant Functional Genomics of the Ministry of Education/Jiangsu Key Laboratory of Crop Genomics and Molecular Breeding/Zhongshan Biological Breeding Laboratory/Jiangsu Co-Innovation Center for Modern Production Technology of Grain Crops, College of Agriculture, Yangzhou University, Yangzhou 225009, China; 2 Shanghai Key Laboratory of Plant Molecular Sciences, College of Life Sciences, Shanghai Normal University, Shanghai 200234, China; 3 International Maize and Wheat Improvement Center (CIMMYT), Mexico D.F. 06600, Mexico; 4 Peking University Institute of Advanced Agricultural Sciences, Weifang, Shandong 261325, China; 5 BGI Bioverse, Shenzhen 518083, China; 6 MolBreeding Biotechnology Co., Ltd., Shijiazhuang 050035, China

**Keywords:** genomic prediction, hybrid, metabolome-wide association studies, metabolic marker, predictive ability

## Abstract

Hybrid breeding is widely acknowledged as the most effective method for increasing crop yield, particularly in maize and rice. However, a major challenge in hybrid breeding is the selection of desirable combinations from the vast pool of potential crosses. Genomic selection (GS) has emerged as a powerful tool to tackle this challenge, but its success in practical breeding depends on prediction accuracy. Several strategies have been explored to enhance prediction accuracy for complex traits, such as the incorporation of functional markers and multi-omics data. Metabolome-wide association studies (MWAS) help to identify metabolites that are closely linked to phenotypes, known as metabolic markers. However, the use of preselected metabolic markers from parental lines to predict hybrid performance has not yet been explored. In this study, we developed a novel approach called metabolic marker-assisted genomic prediction (MM_GP), which incorporates significant metabolites identified from MWAS into GS models to improve the accuracy of genomic hybrid prediction. In maize and rice hybrid populations, MM_GP outperformed genomic prediction (GP) for all traits, regardless of the method used (genomic best linear unbiased prediction or eXtreme gradient boosting). On average, MM_GP demonstrated 4.6% and 13.6% higher predictive abilities than GP for maize and rice, respectively. MM_GP could also match or even surpass the predictive ability of M_GP (integrated genomic-metabolomic prediction) for most traits. In maize, the integration of only six metabolic markers significantly associated with multiple traits resulted in 5.0% and 3.1% higher average predictive ability compared with GP and M_GP, respectively. With advances in high-throughput metabolomics technologies and prediction models, this approach holds great promise for revolutionizing genomic hybrid breeding by enhancing its accuracy and efficiency.

## Teaser

This study reports a metabolic marker-assisted genomic prediction strategy, that incorporates significant metabolite variants from parental lines to improve the accuracy of hybrid prediction. Compared with traditional approaches, this method enables more precise and effective selection for desirable traits.

## Introduction

Hybrid breeding has proved to be the most efficient approach for increasing yield potential in various crops, notably maize and rice (Tu et al., 2000; Duvick, 2001). However, selection of the optimum combinations from a wide range of potential crosses presents a great challenge in hybrid breeding. Genomic selection (GS) has emerged as a solution to this challenge, using genome-wide markers to predict the genomic values of individuals before phenotyping (Meuwissen et al., 2001; Hickey et al., 2014). Genomic hybrid breeding, a special form of GS, leverages markers derived from parental lines to predict hybrid performance, thereby reducing breeding cycles and enhancing genetic gain (Xu et al., 2014; Crossa et al., 2017; Cui et al., 2020). Several studies have confirmed the effectiveness of genomic hybrid breeding (Technow et al., 2014; Zhao et al., 2015; Yang et al., 2022). The success of GS in practical breeding largely depends on the accuracy of genomic prediction (GP) (Xu et al., 2021a). Despite the availability of whole-sequence information, GS may not fully capture the intricate interactions among genes and their downstream regulation, which are integral to the entire process linking genotype to phenotype (Westhues et al., 2017; Hu et al., 2019). For complex quantitative traits, particularly those heavily influenced by environmental factors, such as grain yield, there exists a bottleneck that hinders the improvement of prediction accuracy (Xu et al., 2020; Resende et al., 2024).

With advances in high-throughput molecular biotechnology, it has become possible to predict phenotypes using metabolomic data. The metabolome serves as a link between genotype and phenotype, offering the potential to enhance predictive abilities compared with genomic data by shedding light on downstream interactions (Washburn et al., 2020). For example, the predictive ability of metabolomic data from parental lines to predict the yield of rice hybrids was nearly twice that of genomic data (Xu et al., 2016). Using 56 110 SNPs and 130 metabolites from 285 maize inbred lines and two testers, the general combining abilities of seven traits in maize were predicted, and the results indicated comparable predictive abilities between the two data types (Riedelsheimer et al., 2012). The integration of multi-omics data is increasingly being explored to further enhance prediction accuracy. The combination of genomic, metabolomic, and transcriptomic data can significantly improve predictive abilities for various agronomic traits across diverse plant species (Hu et al., 2021; Wu et al., 2022), highlighting the potential of integrating genomic and metabolomic data to enhance genomic prediction accuracy.

The incorporation of prior or preselected biological information into GP models is another viable approach to enhance prediction accuracy. For instance, the integration of GWAS findings into genomic best linear unbiased prediction (GBLUP) resulted in a 4.8% improvement in the prediction of loin muscle area in pigs (Liu et al., 2023a). Similarly, the use of single-nucleotide polymorphisms (SNPs) preselected from whole-genome sequencing (WGS) data on the basis of expression quantitative trait locus mapping of all genes led to better predictive abilities for startle responses in fruit flies compared with the use of WGS data alone (Ye et al., 2020). In rice, the GS + *de novo* GWAS strategy outperformed six other models in a tropical breeding population across several traits and environments (Spindel et al., 2016). Together, these studies suggest that the integration of prior or preselected biological information can further enhance the accuracy of GS.

Previous studies have demonstrated the effectiveness of metabolome-wide association studies (MWAS) in identifying metabolic markers, i.e. metabolites that are closely linked to phenotypes (Gamboa-Becerra et al., 2019; Xu et al., 2021b). Because of the high dimensionality, noise, and variability in metabolomics data, the identification of metabolic markers is challenging. Current methods for the detection of metabolic markers include partial least-squares discriminant analysis, orthogonal partial least-squares discriminant analysis, artificial neural networks, support vector machines, and other multivariate analysis methods (Worley and Powers, 2013). In a study involving 368 maize inbred lines, 43 metabolites significantly associated with 100-kernel weight were identified using stepwise regression (Wen et al., 2014). Using an improved least absolute shrinkage and selection operator (LASSO) method, 15 metabolites significantly associated with six agronomic traits were identified in 339 maize inbred lines (Xu et al., 2017). A simulation study indicated that the LASSO method had the highest power and lowest false-positive rate among four MWAS methods, detecting 25 metabolites significantly associated with yield-related traits in 533 rice varieties (Wei et al., 2018). These metabolic markers directly influence phenotypic traits, reflecting immediate physiological status and environmental interactions, and are thus expected to provide more accurate predictions. However, the integration of such preselected biological information into GS remains to be explored.

In this study, we developed a novel approach called metabolic marker-assisted GP (MM_GP), which incorporates significant metabolites identified from parental lines by MWAS into GS models to improve the accuracy of hybrid prediction. The performance of MM_GP was evaluated using 425 maize hybrids derived from 205 inbred lines and 278 rice hybrids from 210 recombinant inbred lines (RILs). The proposed MM_GP approach offers a distinct advantage in refining GP, facilitating more precise and effective selection for desirable traits in crop hybrid breeding.

## Results

### Metabolite profiling of seedling leaves in maize inbred lines

**Figure 1:**
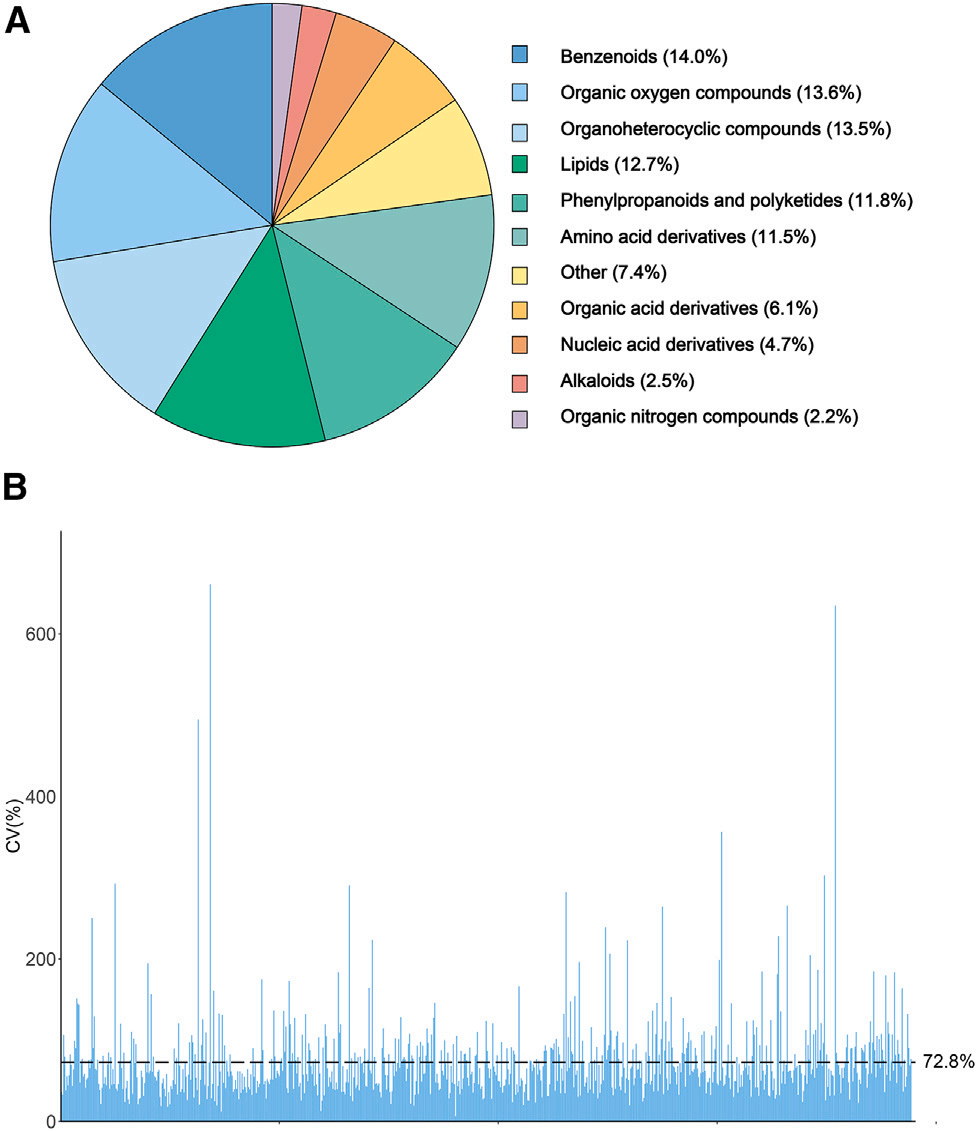
Metabolic profiling of 777 metabolites from 205 maize inbred lines. **(A)** Classification of 777 metabolites. **(B)** Distribution of the coefficients of variation (CVs) of 777 metabolites.

Using a non-targeted liquid chromatography–mass spectrometry (LC–MS) method, 925 metabolites were identified from the seedling leaves of 205 maize inbred lines, each with two biological replicates. After excluding metabolites with significantly different concentrations (*p* < 0.01) between replicates, 777 metabolites remained. Among these metabolite features, 557 were annotated and classified into 11 categories (Figure 1A and Supplemental Table 1). The three most abundant categories were benzenoids (14.0%), organic oxygen compounds (13.6%), and organoheterocyclic compounds (13.5%). Levels of metabolite accumulation varied substantially among the inbred lines, with an average coefficient of variation (CV) of 72.8%. A majority of the metabolites (66.0%) exhibited a CV of >50%, particularly the benzenoids (Figure 1B and Supplemental Table 1).

### Identification of metabolic markers that influence agronomic traits in maize

**Figure 2:**
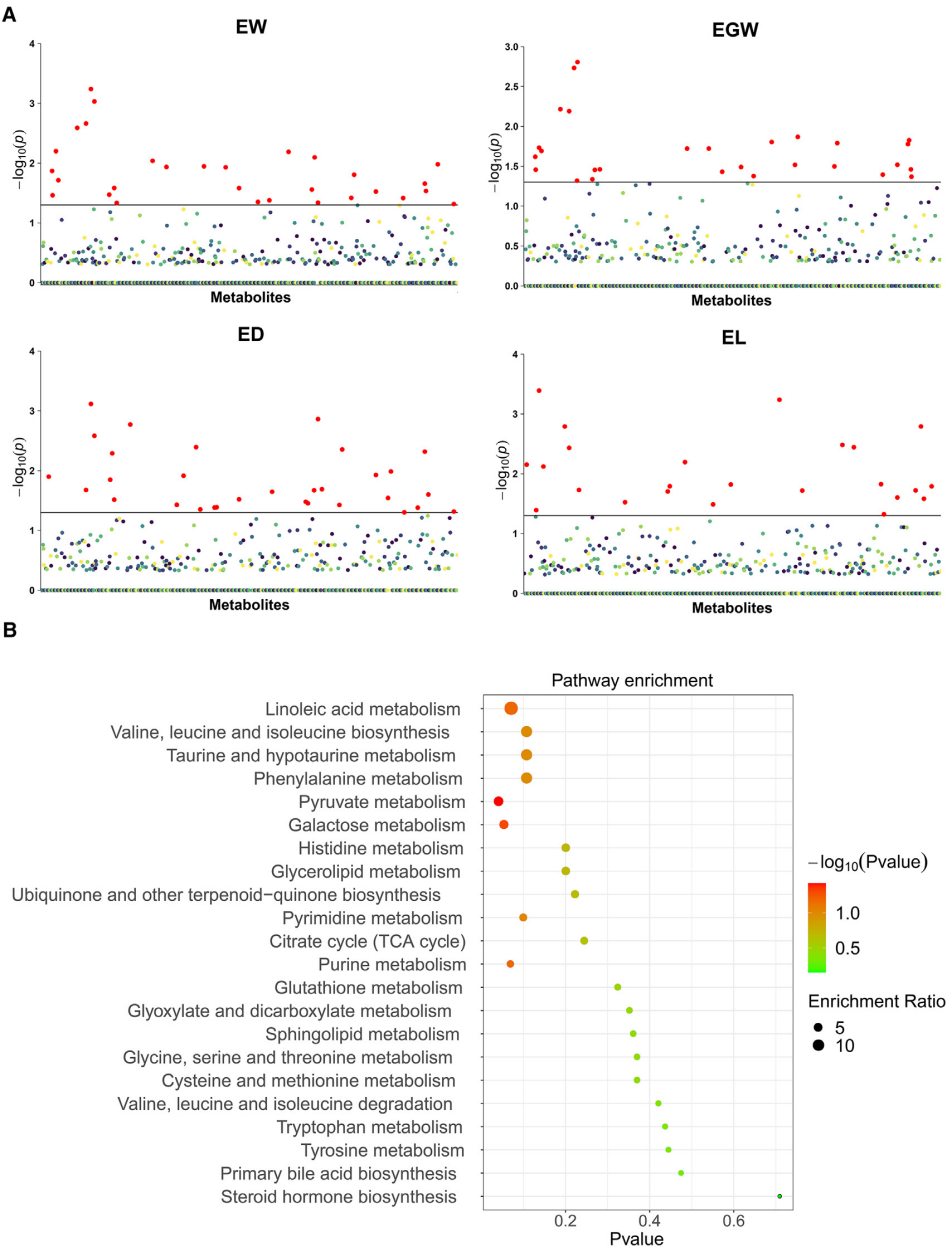
Identification of metabolites associated with four traits in maize. **(A)** Metabolites significantly associated with four traits of 205 maize inbred lines. The horizontal black lines represent the critical values at the 0.05 significance level. **(B)**Enriched pathways of metabolic markers.

Using the LASSO method, 78 significant metabolites were identified in maize inbred lines by MWAS: 30, 28, 31, and 24 metabolites for ear weight (EW), ear grain weight (EGW), ear diameter (ED), and ear length (EL), respectively (Figure 2A and Supplemental Table 2). Forty-seven of the identified metabolites were annotated and classified into 10 categories, with benzenoids (17.0%), organic oxygen compounds (14.8%), and phenylpropanoids and polyketides (14.8%) being the most numerous. In addition, 28, six, and one metabolites showed significant associations with two, three, and four traits, respectively (Supplemental Table 3). For instance, metabolite m863 (salicylic acid) exhibited significant correlations with both EW and EGW. Metabolite m36 (leucine) had significant associations with EW, EGW, and EL, and metabolite m111 (taurine) was significantly associated with all four traits.

The percentage of phenotypic variation explained depended on traits and metabolic markers, ranging from 1.0% to 6.0% (Supplemental Table 2). Metabolite m126 (hypoxanthine) explained the most phenotypic variation for EW and ED, and m136 (valeric acid) and m36 (leucine) were the top contributors to EGW and EL, respectively. Functional enrichment analysis was performed on the 47 annotated metabolic markers, resulting in the identification of 22 enriched metabolic pathways. The top five pathways were pyruvate metabolism, galactose metabolism, linoleic acid metabolism, purine metabolism, and pyrimidine metabolism (Figure 2B and Supplemental Table 4). Notably, the enrichment of pyruvate metabolism reached a significant level.

### Evaluation of MM_GP for hybrid prediction in maize

**Figure 3:**
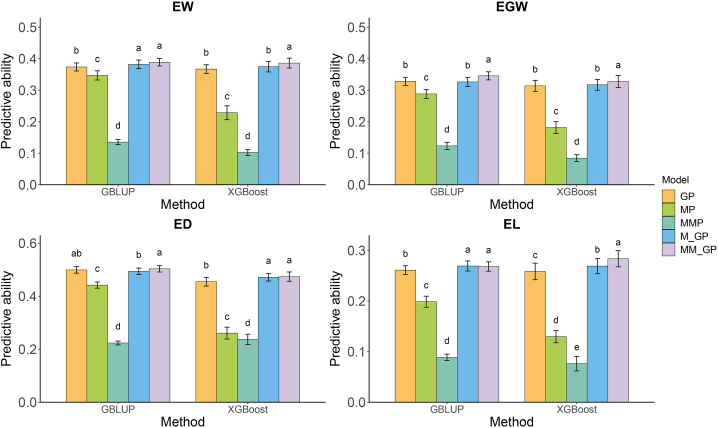
Predictive abilities for four traits in 425 maize hybrids obtained from five prediction models using GBLUP and XGBoost methods. The four traits are ear weight (EW), ear grain weight (EGW), ear diameter (ED), and ear length (EL). The five prediction models are GP, MP, MMP, M_GP, and MM_GP, representing genomic prediction, metabolomic prediction, metabolic marker prediction, integrated genomic–metabolomic prediction, and metabolic marker-assisted genomic prediction, respectively. In each histogram, different lowercase letters above the bars indicate significant differences (*p* < 0.05) between the models.

To examine the capacity of MM_GP for hybrid prediction in maize, we compared the predictive abilities of five prediction models: GP, metabolomic prediction (MP), metabolic marker prediction (MMP), integrated genomic-metabolomic prediction (M_GP), and metabolic marker-assisted GP (MM_GP). Metabolites that showed significant associations with the target trait were considered to be metabolic markers and were used in MMP and MM_GP. The predictive abilities from 10-fold cross-validation with 20 repetitions varied from 0.259 to 0.499 for GP, 0.130 to 0.442 for MP, 0.076 to 0.237 for MMP, 0.269 to 0.494 for M_GP, and 0.268 to 0.503 for MM_GP across the four agronomic traits tested (Figure 3). Among these traits, prediction performance was highest for ED, followed by EW, EGW, and EL. Among the models, MP and MMP exhibited the worst prediction performance. MM_GP displayed better predictive abilities than GP. Specifically, with GBLUP, MM_GP improved the predictive ability for EW by 4.1%, EGW by 5.3%, ED by 0.8%, and EL by 2.7%. Similarly, with eXtreme gradient boosting (XGBoost), MM_GP increased predictive ability for EW by 5.2%, EGW by 4.4%, ED by 4.2%, and EL by 9.7%.

The predictive ability of MM_GP also matched or even exceeded that of M_GP. When using GBLUP, MM_GP increased predictive ability by 1.8% for EW, 5.9% for EGW, and 1.8% for ED compared with M_GP, although their predictive abilities for EL were similar. When using XGBoost, MM_GP increased predictive ability by 3.0% for EW, 3.3% for EGW, 0.5% for ED, and 5.4% for EL compared with M_GP. Notably, M_GP did not improve the predictive ability for some traits compared with GP, whereas MM_GP did. For example, in the case of EGW with GBLUP, M_GP decreased predictive ability by 0.6% compared with GP, whereas MM_GP increased it by 5.3%. Overall, MM_GP consistently performed the best among the five models, regardless of the method used (GBLUP or XGBoost).

**Figure 4:**
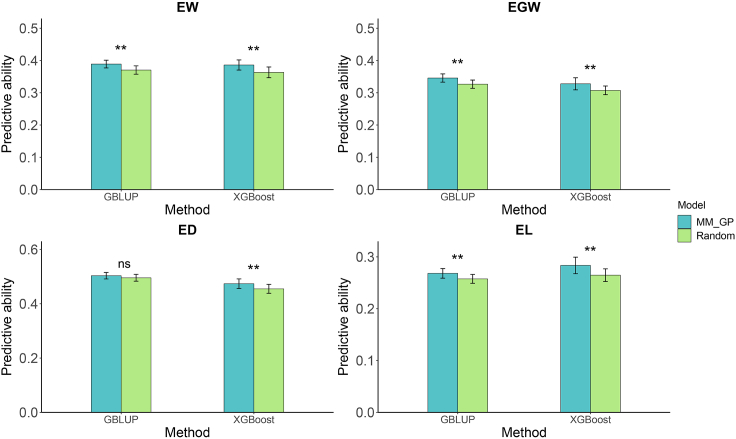
Predictive abilities for four traits in hybrid maize obtained from integrated genomic data and randomly selected metabolites using GBLUP and XGBoost methods. The number of randomly selected metabolites corresponds to the number of metabolic markers. ∗∗*p* < 0.01.

To determine whether the enhanced predictive ability of MM_GP was attributable to the small number of metabolic markers, we randomly selected an equal number of metabolites from the metabolomic data to match the number of metabolic markers. Across an average of 10 replicated samples, the predictive abilities of the randomly selected metabolites for assisting in GP were significantly lower than those of MM_GP (Figure 4). Specifically, using GBLUP, the randomly selected metabolites resulted in a significant decrease in predictive ability for EW, EGW, ED, and EL by 4.8%, 5.8%, 1.4%, and 4.1%, respectively, compared with MM_GP. Similarly, with XGBoost, the randomly selected metabolites significantly reduced predictive ability for EW, EGW, and EL by 6.3%, 6.6%, and 7.1%, respectively. Therefore, we conclude that the improved predictive ability of MM_GP cannot be attributed solely to the small number of metabolic markers.

### Integration of shared significant metabolic markers in MM_GP

**Figure 5:**
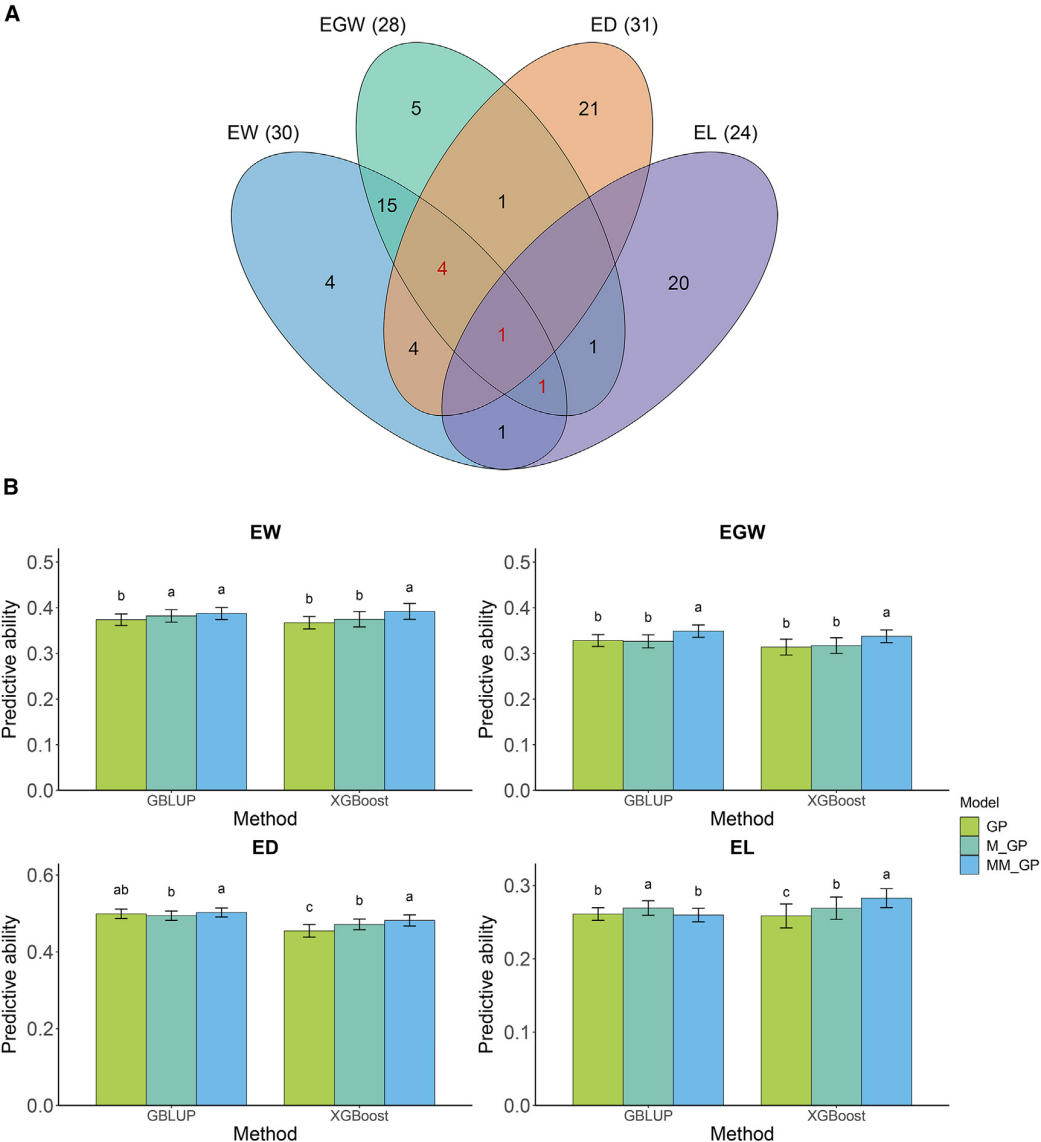
Metabolites significantly associated with three or more traits in maize. **(A)** The number of metabolites significantly associated with four traits of 205 maize inbred lines. The red font indicates the numbers of metabolites significantly associated with three or more traits. **(B)** Predictive abilities for four traits in hybrid maize obtained from MM_GP using GBLUP and XGBoost methods with metabolic markers identified from the parental lines. In each histogram, different lowercase letters above the bars indicate significant differences (*p* < 0.05) between the models.

Six metabolites were found to be significantly associated with three or more traits (Figure 5A). To test the contribution of these shared significant metabolic markers to GP, we combined them with genomic data to predict the four traits in hybrid maize (Figure 5B). The predictive abilities using GBLUP were 0.387 (EW), 0.349 (EGW), 0.502 (ED), and 0.260 (EL), and those using XGBoost were 0.392 (EW), 0.338 (EGW), 0.482 (ED), and 0.283 (EL). MM_GP, which integrated the six shared metabolic markers, showed greater predictive ability than GP and M_GP. Compared with GP, MM_GP with GBLUP significantly increased predictive ability by 3.6% for EW and 6.3% for EGW, although their predictive abilities for ED and EL were similar. Likewise, MM_GP with XGBoost significantly increased predictive ability by 6.8% for EW, 7.6% for EGW, 6.0% for ED, and 9.4% for EL. Compared with M_GP, MM_GP with GBLUP significantly increased predictive ability by 6.9% for EGW and 1.7% for ED, and MM_GP with XGBoost significantly increased predictive ability by 4.6% for EW, 6.4% for EGW, 2.2% for ED, and 5.1% for EL. These findings highlight the greater potential of MM_GP to improve the accuracy of genomic hybrid prediction compared with other methods.

### Evaluation of MM_GP for hybrid prediction in rice

**Figure 6:**
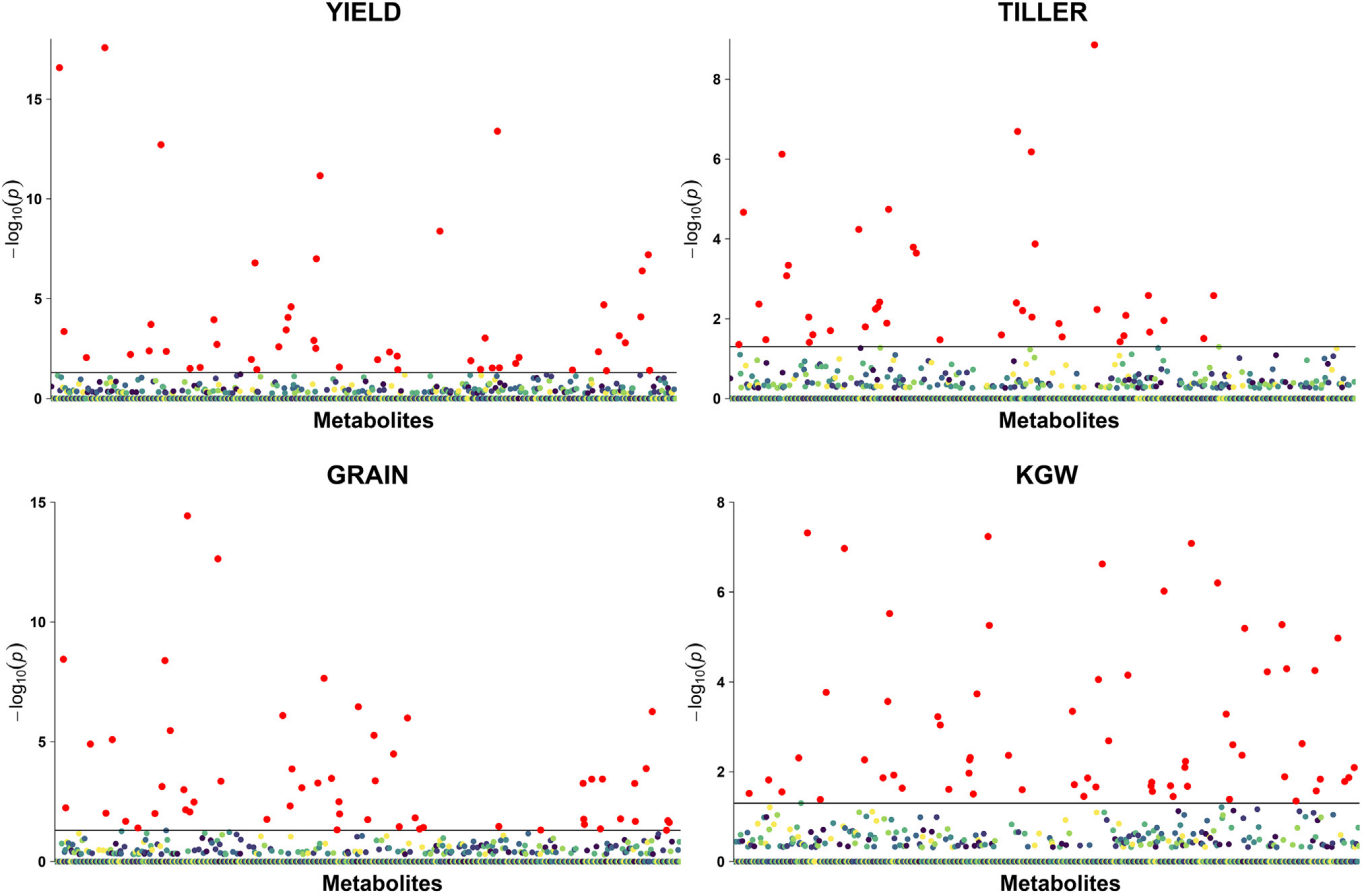
Metabolites significantly associated with four traits in 210 rice RILs. The horizontal black lines represent the critical values at the 0.05 significance level.

To confirm the advantages of MM_GP observed in maize, we performed a similar analysis in rice. Using the LASSO method, we detected 171 metabolites significantly associated with four traits in rice RIL populations: 48 for yield per plant (YIELD), 40 for tiller number per plant (TILLER), 55 for grain number per panicle (GRAIN), and 64 for 1000-grain weight (KGW) (Figure 6 and Supplemental Table 5). Among these metabolites, 138 were significantly associated with one trait, 30 with two traits, and three with three traits (Supplemental Table 6). For example, metabolite m0149-L (sn-glycero-3-phosphocholine) was significantly associated with only one trait (YIELD), m0092-L (D-pantothenic acid) with two traits (YIELD and GRAIN), and m0643-L (chrysoeriol C-hexoside derivative) with three traits (YIELD, GRAIN, and KGW). No metabolites were significantly associated with all the tested traits.

**Figure 7:**
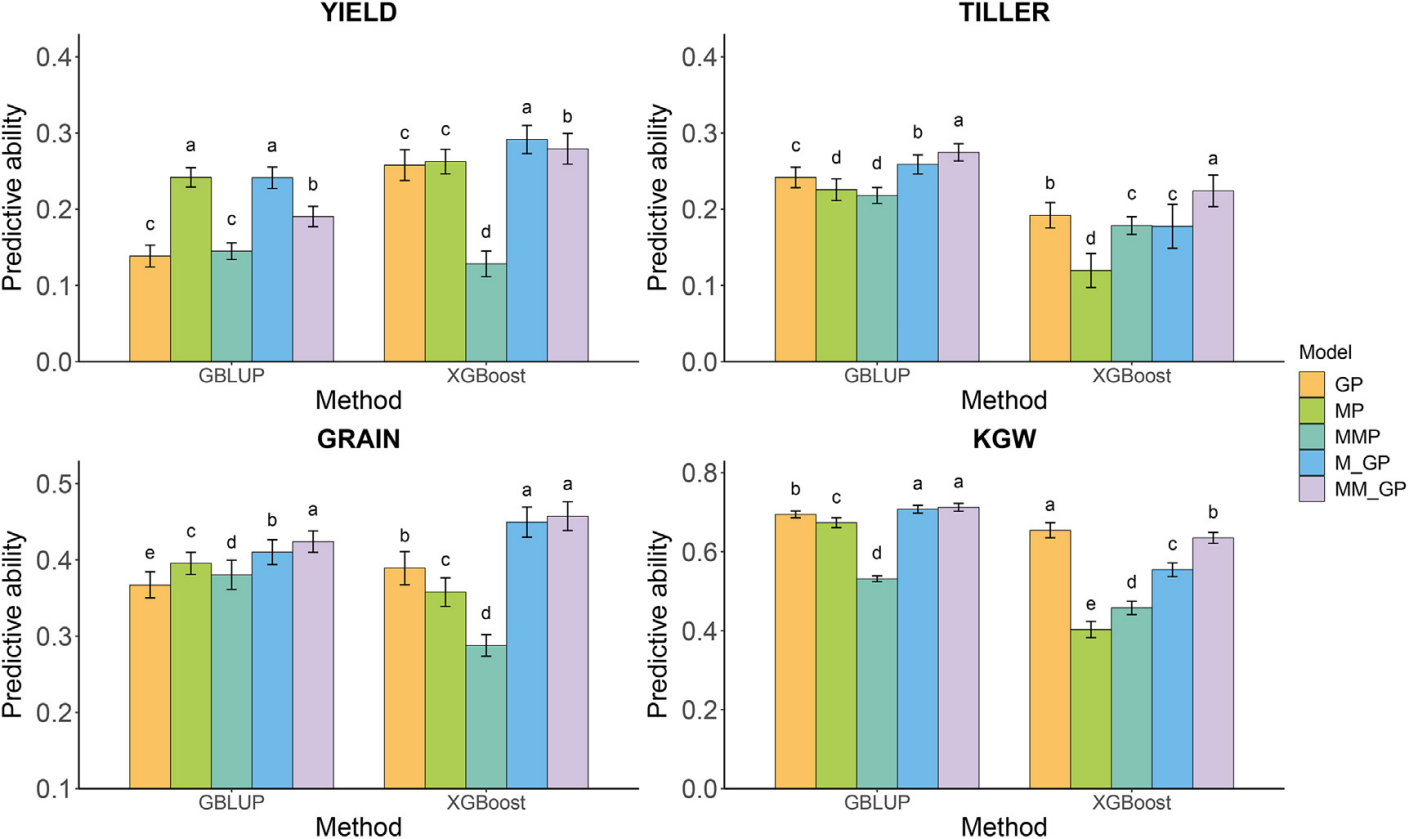
Predictive abilities for four traits in 278 rice hybrids obtained from five prediction models using GBLUP and XGBoost methods. The four traits are yield per plant (YIELD), tiller number per plant (TILLER), grain number per panicle (GRAIN), and 1000-grain weight (KGW). The five prediction models are GP, MP, MMP, M_GP, and MM_GP. In each histogram, different lowercase letters above the bars indicate significant differences (*p* < 0.05) between the models.

We next examined the predictive abilities of the five aforementioned models for four traits in hybrid rice (Figure 7). Predictive abilities varied from 0.138 to 0.694 for GP, 0.120 to 0.673 for MP, 0.128 to 0.531 for MMP, 0.178 to 0.707 for M_GP, and 0.190 to 0.712 for MM_GP across the four agronomic traits. MM_GP and M_GP performed well for most traits, whereas MMP performed poorly. Comparison of the predictive abilities of GP and MM_GP for the four traits in hybrid rice yielded results consistent with those in maize. Using GBLUP, MM_GP demonstrated significantly higher predictive ability for YIELD (by 37.5%), TILLER (13.6%), GRAIN (15.4%), and KGW (2.6%) compared with GP. Using XGBoost, MM_GP significantly outperformed GP for three traits: YIELD (by 8.3%), TILLER (16.7%), and GRAIN (17.5%). MM_GP also outperformed M_GP in the prediction of TILLER, GRAIN, and KGW. Using GBLUP, MM_GP exhibited significantly higher predictive ability for TILLER (by 6.1%) and GRAIN (3.4%). Using XGBoost, MM_GP exhibited significantly higher predictive ability for TILLER (by 26.2%) and KGW (14.5%). On average, MM_GP increased predictive ability by 3.4% (relative to M_GP), 13.6% (relative to GP), and 24.1% (relative to MP) across all traits and methods. These findings demonstrate the greater potential of MM_GP in hybrid rice compared with other tested methods.

We then compared the predictive ability of metabolic markers with that of an equivalent number of randomly selected metabolites and observed results similar to those found in maize (Supplemental Figure 1). Specifically, using GBLUP, the randomly selected metabolites significantly reduced the predictive ability for YIELD, TILLER, GRAIN, and KGW by 4.8%, 10.0%, 9.4%, and 1.8%, respectively, compared with MM_GP. Similarly, using XGBoost, the randomly selected metabolites significantly reduced the predictive ability for YIELD, TILLER, GRAIN, and KGW by 6.9%, 27.5%, 24.0%, and 2.9%. We also analyzed the metabolites in two tissues, flag leaves and germinated seeds, and evaluated the MM_GP model separately for these two tissues (designated MM_GP_leaf and MM_GP_seed). Predictive ability ranged from 0.201 to 0.717 for MM_GP_leaf and from 0.158 to 0.704 for MM_GP_seed across the four traits (Supplemental Figure 2). Notably, MM_GP_leaf exhibited a higher predictive ability than MM_GP_seed. Using GBLUP, MM_GP_leaf demonstrated significantly greater predictive ability for YIELD (by 27.4%), TILLER (17.8%), GRAIN (11.0%), and KGW (1.9%) compared with MM_GP_seed. Using XGBoost, MM_GP_leaf significantly outperformed MM_GP_seed for TILLER (by 21.9%) and GRAIN (24.9%).

### Predicting untested crosses using MM_GP

Using parameters estimated from the training sample, we predicted EW for all 20 910 potential hybrids in maize and YIELD for 21 945 potential hybrids in rice using the MM_GP model. The average predicted values of the top 100 crosses were significantly higher than those of the bottom 100 crosses for both EW and YIELD (Supplemental Tables 7 and 8). When GBLUP was used, the average predicted values of the top 100 crosses for EW and YIELD increased by 62.7% and 48.8%, respectively, compared with the average predicted phenotypic values of the bottom 100 crosses. Similarly, when XGBoost was used, the average predicted values of the top 100 crosses for EW and YIELD rose by 60.5% and 50.4%, respectively, compared with the average predicted phenotypic values of the bottom 100 crosses. Supplemental Figures 3 and 4 illustrate the average predicted phenotypic values of EW and YIELD when selecting the top crosses for hybrid breeding. For instance, if the top 10 crosses predicted by XGBoost were used for hybrid breeding, the average predicted EW and YIELD of these crosses would be 198.27 and 51.15, respectively, indicating gains of 26.6% and 17.9% in EW and YIELD. If the top 10 crosses predicted by GBLUP were used for hybrid breeding, the average predicted values would be 198.36 for EW and 52.11 for YIELD, reflecting gains of 26.4% and 19.6% in EW and YIELD, respectively.

## Discussion

In this study, we propose an innovative approach, MM_GP, which first integrates metabolic markers from parental lines with GS models to predict hybrid performance in maize and rice populations. Our findings indicate that incorporating a small proportion of selected metabolic markers enhances the accuracy of GP. Compared with conventional GP models, the integration of metabolomic data resulted in higher predictive abilities for maize (1.8%) and rice (12.6%), and the integration of selected metabolic markers increased predictive abilities further (4.6% for maize and 13.6% for rice), highlighting the potential of leveraging metabolic data to predict yield-related traits. This result may be due to the additional genetic information implicitly captured by metabolites. Whereas GP models focus on genetic variations at the gene level, M_GP and MM_GP are capable of capturing a broader spectrum of genetic variation and physiological epistasis (Fernie and Schauer, 2009; Feher et al., 2014; Guo et al., 2016; Wang et al., 2021b).

Integration of selected metabolic markers has shown promise in enhancing predictive abilities, potentially surpassing the integration of entire metabolomic data. Our analysis indicated that the MM_GP model generally exhibited superior predictive abilities compared with the M_GP model in maize and rice populations. Notably, the integration of only six selected metabolic markers significantly associated with multiple traits resulted in 3.1% higher predictive ability compared with the M_GP model in maize. This improvement may be attributed to the benefits of feature selection (Xu et al., 2022). Feature selection not only reduced overfitting in the MLR algorithm but also significantly improved the predictive ability of the GLM algorithm for rapeseed seed yield (Shahsavari et al., 2023). In Chinese Holsteins, the use of regularized regression models for feature selection of WGS data demonstrated that combining preselected SNPs with 50K SNP chip data could improve the predictive abilities for milk, protein, and fat yields compared with WGS data and 50K SNP chip data alone (Li et al., 2022). In our study, the identification of metabolic markers via MWAS enabled feature selection of metabolomic data, potentially aiding in the elimination of irrelevant or redundant features, preventing overfitting, and enhancing model generalization.

The improved predictive ability of MM_GP might also be attributed to the incorporation of prior biological information. This assertion is supported by a comparison of the predictive performance of selected metabolic markers with an equivalent number of randomly selected metabolites. Through integration of GWAS results from public databases, GS accuracy increased for two out of three traits in a dairy cattle dataset and nine out of 11 traits in a rice dataset (Zhang et al., 2014). The inclusion of significant SNPs from GWAS improved the prediction accuracy of GS models for 1000-grain weight and amylose content in hybrid rice (Yu et al., 2022) and for nine agronomic traits by 4.0%–19.9% in rice (Zhang et al., 2023). Selection of optimal marker sets and prediction of phenotypes in rice and soybean data using the GMStool developed for GWAS analysis demonstrated higher prediction accuracy than using all SNP markers (Jeong et al., 2020). Other studies also showed that integration of prior GWAS information enhanced predictive ability in livestock species and traits, such as live weight in alpine merino sheep (Li et al., 2023), milk fatty acid composition in dairy cattle (Gebreyesus et al., 2019), and multiple traits in Hanwoo beef cattle (de Las Heras-Saldana et al., 2020). These studies underscore the advantages of incorporating existing biological knowledge at the DNA level. Our results suggest that leveraging prior information at the metabolite level can improve predictive ability in maize and rice, offering potential for wider applications across diverse populations and crop species.

The improved predictive ability of MM_GP relative to GP was significantly greater in rice, with an increase of up to 13.6%, compared with a 4.6% improvement in maize. This discrepancy may stem from the tissues used for metabolite analysis and the timing of sample collection (Westhues et al., 2017). In maize, the predictive ability for 100-grain weight in tropical and subtropical environments using metabolites from mature seeds was comparable to that using genomic data, as metabolites in mature seeds are directly linked to yield (Guo et al., 2016). In our study, maize metabolomic data were obtained from seedling leaves in a climate chamber, whereas rice metabolomic data were obtained from flag leaves and germinated seeds, which are more relevant to yield traits. The instability of metabolites in phenotype prediction arises from the dynamic nature of metabolic profiles. Characteristic-level perturbations in metabolites are significantly greater than those in genomic sequences or marker data and are susceptible to variations in sampling conditions, as well as the age and type of tissue (Schrag et al., 2018). Therefore, to enhance prediction accuracy effectively, it is crucial to be explicit about the time points or tissues being sampled. Our study focused on maize metabolomic data collected from seedlings in climate chambers to minimize the impact of environmental fluctuations compared with field conditions. Previous studies have shown the viability of using metabolic profiles obtained from 3.5-day-old roots cultivated in climate chambers for prediction of hybrid performance (de Abreu e Lima et al., 2017). The use of metabolomics in hybrid breeding can benefit from sampling seedlings under controlled conditions, enabling year-round evaluation with available parental lines and simultaneous sampling of multiple tissues such as leaves and roots. The shorter cultivation period leads to more rapid availability of prediction results when developing superior hybrids for further testing (Schrag et al., 2018). Although metabolites in tissues at later developmental stages, such as mature seeds, are associated with yield-related traits, time and resource costs must also be considered. Early-stage sampling under controlled conditions facilitates early selection, thereby reducing breeding cycles and enhancing annual genetic gain.

We also used MM_GP to predict the phenotypic values of 20 910 potential hybrids for EW in maize. The genotypes and metabolites of these future hybrids are not directly measured; instead, they are inferred from their parental lines. The top crosses can be immediately used and transformed into high-performing hybrids. In addition, selection of the top 100 crosses for EW results in gains of 192.24−156.96=35.28±2.68 and 191.68−156.66=35.02±2.69 g per plant when using GBLUP and XGBoost, respectively. Although the improvement in predictive ability of MM_GP in maize appears modest, the gains of 35.28/156.96=22.5% and 35.02/156.66=22.4% achieved through selection of the top 100 hybrids using GBLUP and XGBoost, respectively, represent a noteworthy accomplishment. Among the top 100 maize crosses, A017/A037 had been designated as Suyu 161, a variety developed by Jiangsu Yanjiang Institute of Agricultural Sciences, China. It is worth noting that 24 and nine crosses exhibited a predicted EW greater than that of A017/A037 when using GBLUP and XGBoost, respectively. These crosses merit further validation and could contribute to the development of new varieties aimed at enhancing maize yield.

In this study, we identified metabolites significantly associated with agronomic traits of maize and rice. The well-predicted metabolic markers exhibited various degrees of correlation, showing a roughly equal distribution of both positive and negative correlations. The correlation coefficients ranged from −0.41 to 0.97 in maize and from −0.71 to 0.98 in rice (Supplemental Tables 9 and 10). A total of 411 significant correlations (*p* < 0.01) were identified in maize compared with 3350 in rice. Notably, significant correlations were observed not only between metabolic markers within the same categories but also between markers from different categories (Supplemental Figures 5 and 6). In addition, in maize, nine metabolic markers were associated with shared metabolic pathways and exhibited either upstream or downstream associations (Supplemental Table 4). For instance, metabolites m819 (S-lactoylglutathione) and m838 (malic acid) are both involved in pyruvate metabolism. Metabolites m126 (hypoxanthine), m893 (inosine), and m98 (deoxyguanosine) are associated with purine metabolism. A literature search and information from the Kyoto Encyclopedia of Genes and Genomes database revealed that, among these metabolites, m819 (S-lactoylglutathione) can be converted to m838 (malic acid) through several pathways (Long et al., 2015; Dafre et al., 2017; Schwörer et al., 2021). Metabolites m893 (inosine) and m126 (hypoxanthine) can be interconverted via laccase domain containing 1 (LACC1) (Svetlana et al., 2022).

By assessing the phenotypic variation explained by parental genotypes for 78 metabolic markers in maize, we found that these markers are influenced by parental genotypes to various degrees. Specifically, parental genotypes explained less than 10% of the phenotypic variation in 16 metabolic markers, between 10% and 50% in 37 metabolic markers, and more than 50% in 25 metabolic markers (Supplemental Table 11). An metabolome-based genome-wide association study (mGWAS) analysis of metabolic markers using the FarmCPU (fixed and random model circulating probability unification) method (Liu et al., 2016), detected a total of 30, 19, 75, and 111 significant (*p* < $4.8\times 10^{-7}$) SNPs corresponding to nine, seven, 13, and 15 metabolite markers for EW, EGW, ED, and EL, respectively (Supplemental Figure 7 and Supplemental Table 12). Notably, four common significant SNPs were identified. SNP_3_16890062 and SNP_3_223717387, both located on chromosome 3, were significantly associated with metabolites m126 (hypoxanthine) and m753 (ortho-hydroxyphenylacetic acid); SNP_1_197177004 was significantly associated with metabolites m706 (parthenin) and m375 (histamine); and SNP_7_120230279 was significantly associated with metabolites m614 and m684. These findings suggest shared genetic control over these metabolites. In summary, our study identified a set of SNPs that regulate significant metabolites associated with maize yield traits. These results will facilitate the functional verification of genes and enhance our understanding of metabolic networks, ultimately contributing to the improvement of maize yield.

Some of these metabolic markers play key roles in various plant growth and development processes, directly or indirectly influencing agronomic traits. For instance, metabolite m838 (malic acid) was significantly correlated with EGW and EL in maize. Previous research also found that malic acid was linked to flag-leaf width in wheat (Shi et al., 2020). Malic acid, an organic acid, plays an essential part in regulating carbon metabolism in plants by linking mitochondrial respiratory metabolism to cytosolic biosynthetic pathways. It has important functions in the tricarboxylic acid cycle and metabolic signaling as well (Shan et al., 2023). Another metabolite, m36 (leucine), was found to be related to EW, EGW, and EL in maize. An association between leucine and heading date has been reported in rice (Li et al., 2019). Leucine has been shown to regulate stress tolerance via the plant’s respiratory system (Pires et al., 2016) and can also serve as a plant growth regulator to increase antioxidant capacity and heat resistance (Liu et al., 2023b). Metabolite m863 (salicylic acid) was found to be associated with maize EW and EGW in the present study, and salicylic acid has also been identified at three developmental stages of wheat, namely grain-filling kernels, mature kernels, and germinating kernels (Yin et al., 2024). Another metabolite, m0021-L (trigonelline), which was associated with yield per plant and grain number per panicle in rice in our analysis, has also shown correlations with grain width (Chen et al., 2016; Wei et al., 2018) and grain length (Li et al., 2019). Trigonelline, an alkaloid, plays an important role in the regulation of cell growth and development (Mazzuca et al., 2000). A study on peanuts suggested that reduction of trigonelline level could enhance peanut yield (Cho et al., 2011). Identification of these metabolites can help to reveal biological networks involving genomic loci, metabolites, and traits, enabling us to better understand the genetic mechanisms that underlie different traits.

Our research demonstrates the distinct advantages of metabolic marker-assisted GP (MM_GP) for hybrid prediction in two staple crops, maize and rice. With advances in high-throughput metabolomics technologies and prediction models, this approach has the potential to transform GS by improving its accuracy and efficiency. It not only accelerates the crop breeding process by enabling early selection but also offers valuable insights for advances in precision breeding.

## Methods

### Maize materials

The maize plant materials consisted of 425 hybrids produced using a sparse partial diallel crossing experiment involving 205 inbred lines that were a subset of a previously described maize panel (Wang et al., 2021a). These maize materials were planted in Yangzhou (119.27° E, 32.36° N) and Taian (116.39° E, 35.83° N) in 2018, following a randomized block design with two rows and two replications. Each row contained 13 plants with a plant spacing of 25 cm and a row spacing of 60 cm. Field management practices, including irrigation, weeding, disease and pest control, and fertilization, were performed according to local plot-trial management guidelines. For each inbred line and hybrid, five maize ears of uniform size were selected for evaluation of four traits: EW, EGW, ED, and EL. The 205 maize inbred lines were genotyped using the genotype-by-sequencing method using fresh young leaves collected during the vegetative growth stage. After filtering SNPs with low allelic frequency (<0.05) and high missing rates (>0.1), 104 011 high-quality SNPs were retained for subsequent analysis. The genotypes of the 425 hybrids were inferred from those of their parents.

### Metabolite analysis by LC–MS

Non-targeted LC–MS was used to analyze metabolites in seedling leaves of 205 maize inbred lines. For each maize material, plump and uniform maize seeds were selected for hydroponic experiments in a climate chamber under controlled conditions. Two biological replicates were established for each material, with 10 plants per replicate. At the three-leaf, one-heart stage, leaves from three plants per replicate were collected for metabolomic analysis. These samples were promptly frozen in liquid nitrogen and transferred to −80°C. Each sample was weighed to 200 mg (±1%) in a 2-ml EP tube with 0.6 ml of methanol (−20°C) containing 4 ppm 2-chlorophenylalanine. The mixture was vortexed for 30 s, followed by grinding in a tissue-grinding machine at 65 Hz for 60 s and ultrasonic crushing at 40 kHz for 30 min. The samples were then centrifuged at 25°C and spun at 12 000 rpm for 10 min. The filtered supernatant (300 μl) was transferred to a sample bottle for LC–MS analysis. Chromatographic separation was performed on a Thermo Vanquish system equipped with an ACQUITY UPLC HSS T3 column (150 × 2.1 mm, 1.8 μm, Waters) maintained at 40°C. The temperature of the autosampler was set to 8°C. The gradient elution conditions are given in Supplemental Tables 13 and 14. The ESI-MSn experiments were performed using a Thermo Q Exactive mass spectrometer with a spray voltage of 3.8 kV in positive mode and −2.5 kV in negative mode. The raw data were converted into mzXML format using ProteoWizard software (version 3.0.8789). The XCMS package in R (version 3.1.3) was used for peak identification, filtration, and alignment. A data matrix containing information on mass-to-charge ratio (m/z), retention time, intensity, and other relevant details was generated. To facilitate comparison of data across different magnitudes, the intensity values were subjected to batch normalization. The identification of metabolites was initially confirmed on the basis of exact molecular weight (with a molecular weight error of ≤30 ppm), followed by analysis of the tandem mass spectrometry fragmentation pattern. The Human Metabolome Database (HMDB) (http://www.hmdb.ca/), METLIN (http://metlin.scripps.edu), MassBank (http://www.massbank.jp/), LipidMaps (http://www.lipidmaps.org), mzCloud (https://www.mzcloud.org), and the Panomix proprietary standard database were used to verify annotations and identify metabolites.

### Statistical analysis of metabolomic data

Statistical significance testing was performed on the concentration of each detected metabolite in the two biological replicates. Metabolites that showed a significant difference (*p* < 0.01) between the two replicates were excluded, leaving 777 metabolites for further analysis. The metabolite concentrations were normalized, and the mean value of the two biological replicates was used for subsequent analysis. The CV was calculated for each metabolite, and the phenotypic variation explained by each metabolic marker was determined by the relevant *r*^2^. The LASSO method (Tibshirani, 1997) was used in an MWAS to identify metabolites significantly associated (*p* < 0.05) with agronomic traits of the parental lines. Specifically, the lassopv/R package was used for LASSO computation (Wang and Michoel, 2017). The *p* value was calculated for each metabolite, and those with a *p* value below 0.05 were considered to be significant metabolites. These metabolites were then integrated into GP models as metabolic markers.

### Rice dataset

The rice datasets consisted of 210 RILs obtained from a cross between two rice varieties (Zhenshan 97 and Minghui 63), along with 278 hybrids formed by random pairing of the 210 RILs (Hua et al., 2003). The genomic data included 1619 bins identified from 270 820 SNPs by sequencing all 210 RILs (Yang et al., 2022). The metabolomic data included 1000 metabolites, with 317 detected in germinated seeds and the remaining 683 detected in flag leaves (Gong et al., 2013). Four agronomic traits were analyzed: YIELD, TILLER, GRAIN, and KGW.

### The MM_GP model

We used two GS methods to demonstrate the effectiveness of MM_GP in maize and rice. The first method, GBLUP, used kinship matrices to represent the genetic relationships among individuals based on a mixed linear model (VanRaden, 2008). The second method, XGBoost, is a machine-learning algorithm capable of capturing non-linear relationships without requiring prior information from potential genetic models (Chen and Guestrin, 2016). Detailed information about model structure and optimization is provided below.

#### GBLUP for MM_GP

The GBLUP model for MM_GP is described as

(Equation 1) $$y=X\beta +Z_{G}\gamma_{G}+A_{M}\gamma_{Ma}+D_{M}\gamma_{Md}+\varepsilon$$

where *y* is an n×1 vector of phenotypic observations of hybrids; *X* is an n×p design matrix for the fixed effect; β is the fixed effect; $Z_{G}$ is an n×g genotype matrix of the hybrids; $A_{M}$ and $D_{M}$ are n×m additive and dominance coding matrices of metabolites, respectively, where $A_{M}=\frac{1}{2}\left(M+F\right)$ and $D_{M}=\frac{1}{2}\left|M-F\right|$; and *M* and *F* represent the matrices of metabolic marker concentrations for male and female parents, respectively. The details of the coding system were described in our previous research (Xu et al., 2021c). $\gamma_{G}$, $\gamma_{Ma}$ and $\gamma_{Md}$ were assumed to follow the normal distributions $\gamma_{G}∼N\left(0,\frac{1}{g}\phi_{G}^{2}\right)$, $\gamma_{Ma}∼N\left(0,\frac{1}{m}\phi_{Ma}^{2}\right)$, and $\gamma_{Md}∼N\left(0,\frac{1}{m}\phi_{Md}^{2}\right)$, respectively, where $\phi_{G}^{2}$, $\phi_{Ma}^{2}$, and $\phi_{Md}^{2}$ are the corresponding polygenic variances, *g* and *m* are the numbers of SNPs and metabolites, and ε is an n×1 vector of residual errors with a normal distribution $N\left(0,\sigma_{\varepsilon }^{2}\right)$. The expectation of *y* is E(y)=Xβ, and the variance–covariance matrix is

(Equation 2) $$\text{var}\left(y\right)=V=\frac{1}{g}Z_{G}Z_{G}^{T}\phi_{G}^{2}+\frac{1}{m}A_{M}A_{M}^{T}\phi_{Ma}^{2}+\frac{1}{m}D_{M}D_{M}^{T}\phi_{Md}^{2}=K_{G}\phi_{G}^{2}+{K_{M}}_{a}\phi_{Ma}^{2}+K_{Md}\phi_{Md}^{2}+I\sigma_{\varepsilon }^{2}$$

where $K_{G}$, $K_{Ma}$, and $K_{Md}$ are kinship matrices for random effects $\gamma_{G}$, $\gamma_{Ma}$, and $\gamma_{Md}$, respectively. The variance components were estimated using the restricted maximum likelihood (Patterson and Thompson, 1971; Yin et al., 2023).

After parameters are estimated from the training set, they can be used to predict the phenotypic values of the test set. Assuming $y_{1}$ is an $n_{1}\times 1$ vector of the phenotypic values in the training set, $y_{2}$ is an $n_{2}\times 1$ vector of the phenotypic values in the testing set, and $n_{1}+n_{2}=n$, where *n* is the size of the entire sample, Formula 1 can be rewritten as

(Equation 3) $$\left[\begin{array}{l} y_{1}\\ y_{2} \end{array}\right]=\left[\begin{array}{l} X_{1}\beta \\ X_{2}\beta \end{array}\right]+\left[\begin{array}{l} Z_{G_{1}}\gamma_{G}\\ Z_{G_{2}}\gamma_{G} \end{array}\right]+\left[\begin{array}{l} A_{M_{1}}\gamma_{Ma}\\ A_{M_{2}}\gamma_{Ma} \end{array}\right]+\left[\begin{array}{l} D_{M_{1}}\gamma_{Md}\\ D_{M_{2}}\gamma_{Md} \end{array}\right]+\left[\begin{array}{l} \varepsilon_{1}\\ \varepsilon_{2} \end{array}\right]$$

The expectation and variance–covariance of *y* can be modified as:

(Equation 4) $$E\left[\begin{array}{l} y_{1}\\ y_{2} \end{array}\right]=\left[\begin{array}{l} X_{1}\beta \\ X_{2}\beta \end{array}\right]$$

(Equation 5) $$\text{var}\left[\begin{array}{l} y_{1}\\ y_{2} \end{array}\right]=\left[\begin{array}{cc} V_{11} & V_{12}\\ V_{21} & V_{22} \end{array}\right]=\left[\begin{array}{cc} K_{G_{11}} & K_{G_{12}}\\ K_{G_{21}} & K_{G_{22}} \end{array}\right]\phi_{G}^{2}+\left[\begin{array}{cc} K_{Ma_{11}} & K_{Ma_{12}}\\ K_{Ma_{21}} & K_{Ma_{22}} \end{array}\right]\phi_{Ma}^{2}+\left[\begin{array}{cc} K_{Md_{11}} & K_{Md_{12}}\\ K_{Md_{21}} & K_{Md_{22}} \end{array}\right]\phi_{Md}^{2}+\left[\begin{array}{cc} I_{n_{1}} & 0\\ 0 & I_{n_{2}} \end{array}\right]\sigma_{\varepsilon }^{2}$$

where the kinship matrices have been partitioned into 2 × 2 blocks. After the parameter vector $\theta =\left[\beta ,\phi_{G}^{2},\phi_{Ma}^{2},\phi_{Md}^{2},\sigma_{\varepsilon }^{2}\right]$ is estimated, the predicted phenotypic values of the testing set can be obtained from the following formula:

(Equation 6) $${\hat{y}}_{2}=E(y_{2}|y_{1})=X_{2}\hat{\beta }+\left(K_{G_{21}}\phi_{G}^{2}+K_{Ma_{21}}\phi_{Ma}^{2}+K_{Md_{21}}\phi_{Md}^{2}\right)V_{11}^{-1}\left(y_{1}-X_{1}\hat{\beta }\right)$$

#### XGBoost for MM_GP

XGBoost, proposed by Chen and Guestrin (2016), is an effective and flexible ensemble machine learning algorithm (Ma et al., 2022). The process of XGBoost for MM_GP involved training on a dataset $D=\left\{\left(X_{i},y_{i}\right)\right\}\left(\left|D\right|=n,X_{i}\in R^{q},y_{i}\in R\right)$ with *n* samples and *q* features, where $y_{i}$ represents the phenotypic observation value of the *i*-th hybrid and $X_{i}=\left[\begin{array}{ccc} Z_{G_{i}} & A_{M_{i}} & D_{M_{i}} \end{array}\right]$ is a 1×q feature vector comprising the genotype vector ($Z_{G_{i}}$), metabolite additive coding vector ($A_{M_{i}}$), and metabolite dominance coding vector ($D_{M_{i}}$) of the *i*-th hybrid. Initially, XGBoost generates predicted values by training a tree on the samples, and subsequent trees are built using the residual errors of the previous tree (Yan et al., 2021). After *K* iterations, the predicted phenotypic value (${\hat{y}}_{i}$) can be expressed as

(Equation 7) $${\hat{y}}_{i}=\sum_{k=1}^{K}f_{k}\left(X_{i}\right),f_{k}\in F$$

where $f_{k}\left(X_{i}\right)$ represents the prediction value of the *k*-th decision tree for the *i*-th individual. The tree-structured Parzen estimator, a Bayesian optimization algorithm, was used to explore the hyperparameter space and optimize the hyperparameters of each trait by minimizing the root-mean-square error (Ozaki et al., 2020). The analysis codes are available on GitHub (https://github.com/171702120/yangxu89-GS2024).

### Assessing the predictive abilities of prediction models

The predictive abilities of different prediction models in maize and rice datasets were evaluated using 10-fold cross-validation. This procedure involved randomly dividing the sample into 10 subsets, with nine used for parameter estimation and one for prediction. This process was repeated until all subsets were predicted. Predictive ability was calculated as the determination coefficient between the observed and predicted phenotypic values. To reduce random errors from sample partitioning, the cross-validation procedure was iterated 20 times, and the average of these iterations was calculated to determine the final predictive ability of the models.

## Funding

This work was supported by grants from the 10.13039/501100012166National Key Research and Development Program of China (2023YFD1202200), the 10.13039/501100001809National Natural Science Foundation of China (32170636, 32061143030, 32261143462, 32100448, 32070558), the Seed Industry Revitalization Project of Jiangsu Province (JBGS[2021]009), the Key Research and Development Program of Jiangsu Province (BE2022343, BE2023336), Jiangsu Province Agricultural Science and Technology Independent Innovation (CX(21)1003), the Shenzhen Science and Technology Program (KQTD202303010928390070), the Hebei Science and Technology Program (215A7612D), the Shanghai Agricultural Science and Technology Innovation Program (T2023204), the Provincial Technology Innovation Program of Shandong, China, Qing Lan Project of Jiangsu Province, Yangzhou University High-end Talent Support Program, and the 10.13039/501100012246Priority Academic Program Development of Jiangsu Higher Education Institutions (PAPD).

## Acknowledgments

No conflict of interest is declared.

## Author contributions

C.X., Yang Xu, and Yunbi Xu designed the research. W.Y., J.Q., K.Z., G.Y., Y.Z., Y.L., R.C., and T.T. performed the research. Xin Wang, Y.J., Xinyi Wang, S.H., and P.L. analyzed the data. Y.X. and W.Y. wrote the paper. Y.X., Z.Y., and C.X. revised the manuscript. All authors read and approved the final manuscript.

## Supplemental information

Data S1. Supplemental Tables 1–14

Document S2. Article plus supplemental information
