# Supplementary material for: Metabolic marker-assisted genomic prediction improves hybrid breeding
Source: Plant Commun. 2024 Nov 29;6(3):101199. doi: 10.1016/j.xplc.2024.101199 (PMC11956108; doi:10.1016/j.xplc.2024.101199)
Supplement: Document S1. Supplemental Figures 1–7 [file mmc1.pdf]

Plant Communications, Volume *6*

Supplemental information

Metabolic marker-assisted genomic prediction improves hybrid breeding

Yang Xu, Wenyan Yang, Jie Qiu, Kai Zhou, Guangning Yu, Yuxiang Zhang, Xin Wang, Yuxin Jiao, Xinyi Wang, Shujun Hu, Xuecai Zhang, Pengcheng Li, Yue Lu, Rujia Chen, Tianyun Tao, Zefeng Yang, Yunbi Xu, and Chenwu Xu

Supplemental information

Metabolic marker-assisted genomic prediction improves hybrid breeding

Yang Xu[a,†], Wenyan Yang[a,†], Jie Qiu[b,†], Kai Zhou[a], Guangning Yu[a], Yuxiang Zhang[a], Xin Wang[a], Yuxin Jiao[a], Xinyi Wang[a], Shujun Hu[a], Xuecai Zhang[c], Pengcheng Li[a], Yue Lu, Rujia Chen, Tianyun Tao, Zefeng Yang[a], Yunbi Xu[d,e,f*], Chenwu Xu [a,*]

[a] Key Laboratory of Plant Functional Genomics of the Ministry of Education/Jiangsu Key Laboratory of Crop Genomics and Molecular Breeding/Zhongshan Biological Breeding Laboratory/Jiangsu Co-Innovation Center for Modern Production Technology of Grain Crops, College of Agriculture, Yangzhou University, Yangzhou 225009, China

[b] Shanghai Key Laboratory of Plant Molecular Sciences, College of Life Sciences, Shanghai Normal University, Shanghai 200234, China

[c] International Maize and Wheat Improvement Center (CIMMYT), Mexico D.F. 06600, Mexico

[d] Peking University Institute of Advanced Agricultural Sciences, Weifang, Shandong 261325, China.

[e] BGI Bioverse, Shenzhen 518083, China

[f] MolBreeding Biotechnology Co., Ltd., Shijiazhuang 050035, China

†These authors contribute equally to this work.

***Correspondence:** Chenwu Xu, Email: cwxu@yzu.edu.cn; Yunbi Xu, Email: yunbi.xu@pku-iaas.edu.cn

Short Summary: Metabolic marker-assisted genomic prediction strategy was developed, by incorporating significant metabolite variants from parental lines to improve the accuracy of hybrid prediction. Compared to the traditional one, this offers a distinct advantage allowing more precise and effective selection for desirable traits.

Supplemental Figures

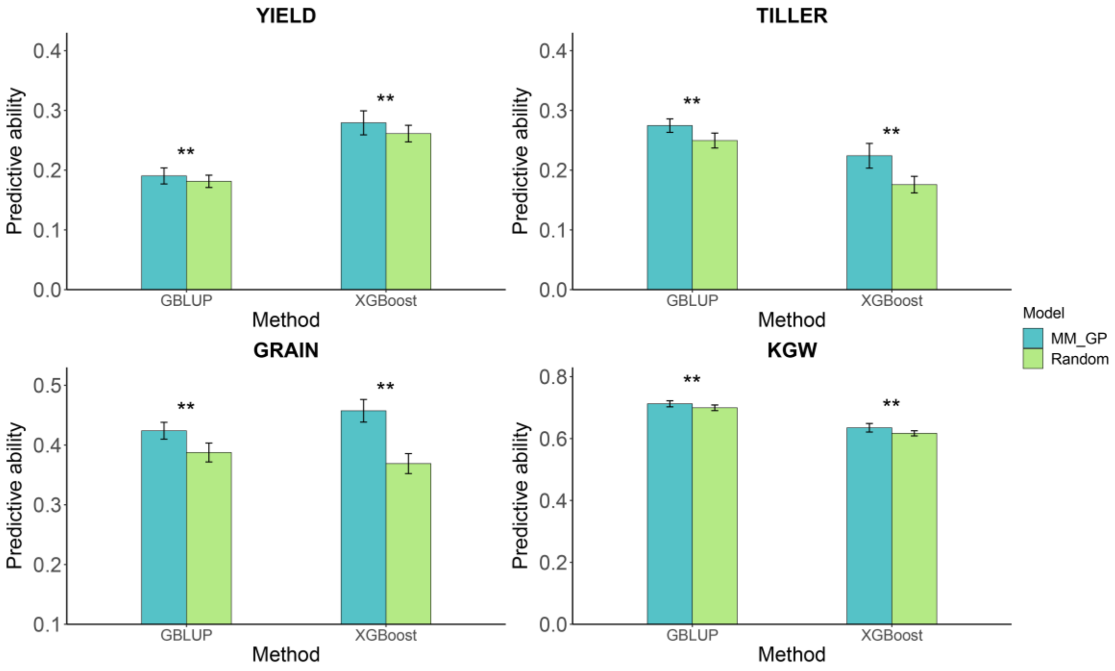

Supplemental Figure 1. Predictive abilities of four traits in hybrid rice obtained from integrated genomic data and randomly selected metabolites (Random), using GBLUP and XGBoost methods. The number of randomly selected metabolites corresponds to the number of metabolic markers. Asterisks (**) indicate a significance level of $P < 0.01$.

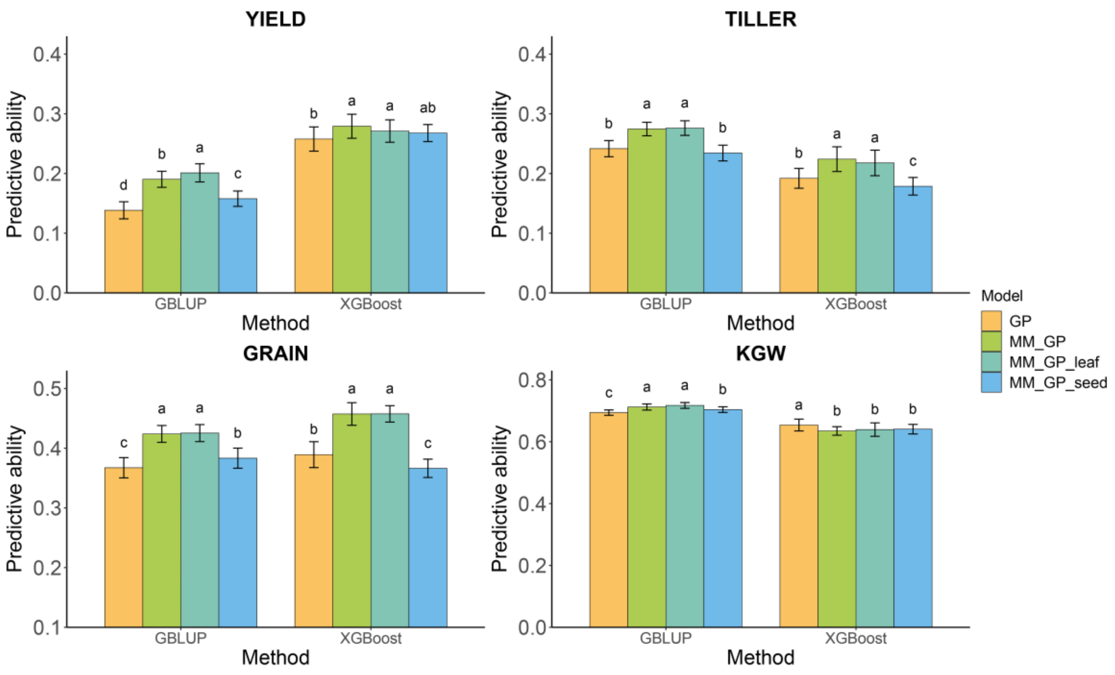

Supplemental Figure 2. The predictive abilities of four traits in hybrid rice were evaluated using the MM_GP model for flag leaves and germinated seeds, referred to as MM_GP_leaf and MM_GP_seed, respectively. In each histogram, distinct lowercase letters positioned above the bars indicate significant differences ($P < 0.05$) between the models.

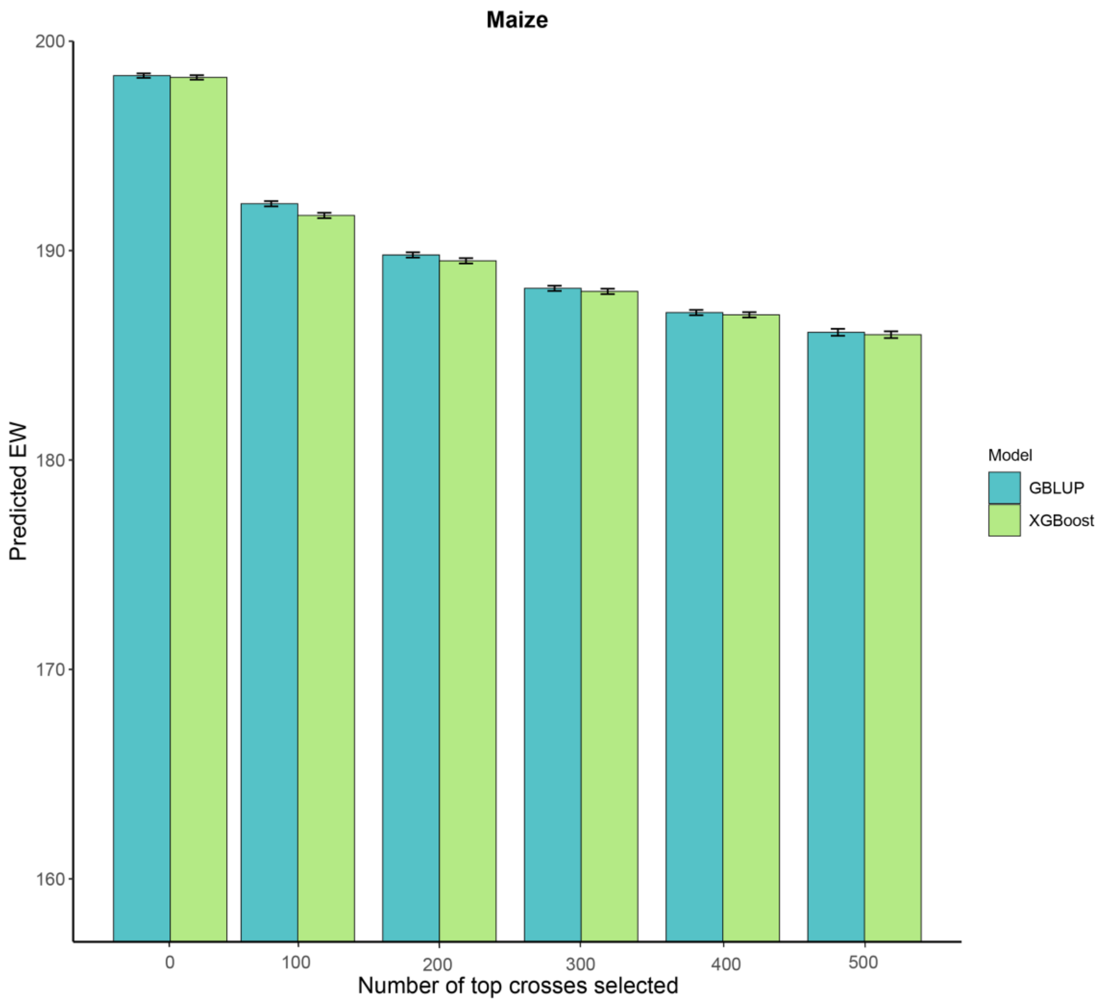

Supplemental Figure 3. Average predicted EW of the top crosses selected for hybrid breeding from MM_GP, utilizing both GBLUP and XGBoost methods. The average EW for all 20,910 potential crosses is 156.96 when using GBLUP and 156.66 when using XGBoost.

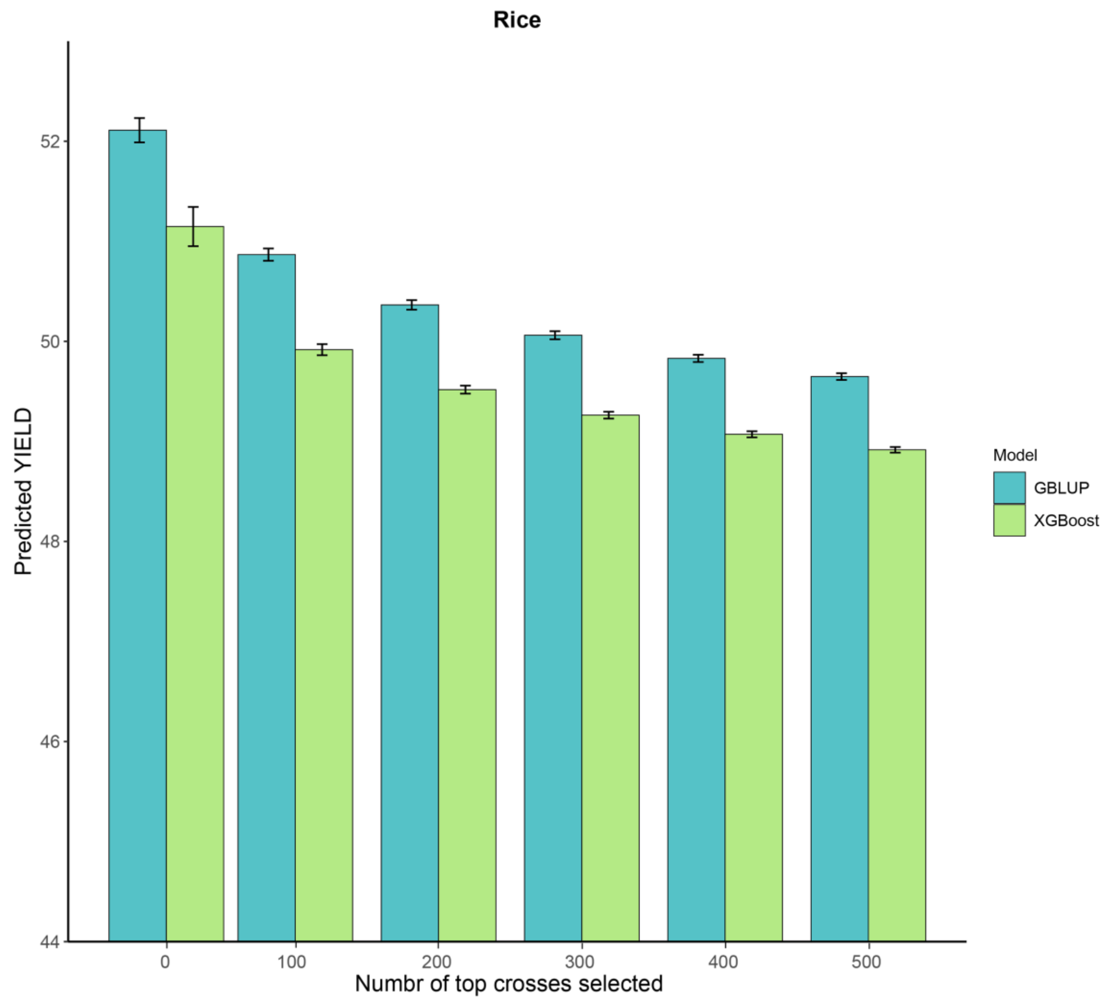

Supplemental Figure 4. Average predicted YIELD of the top crosses selected for hybrid breeding from MM_GP, utilizing both GBLUP and XGBoost methods. The average YIELD for all 21,945 potential crosses is 43.59 when using GBLUP and 43.39 when using XGBoost.

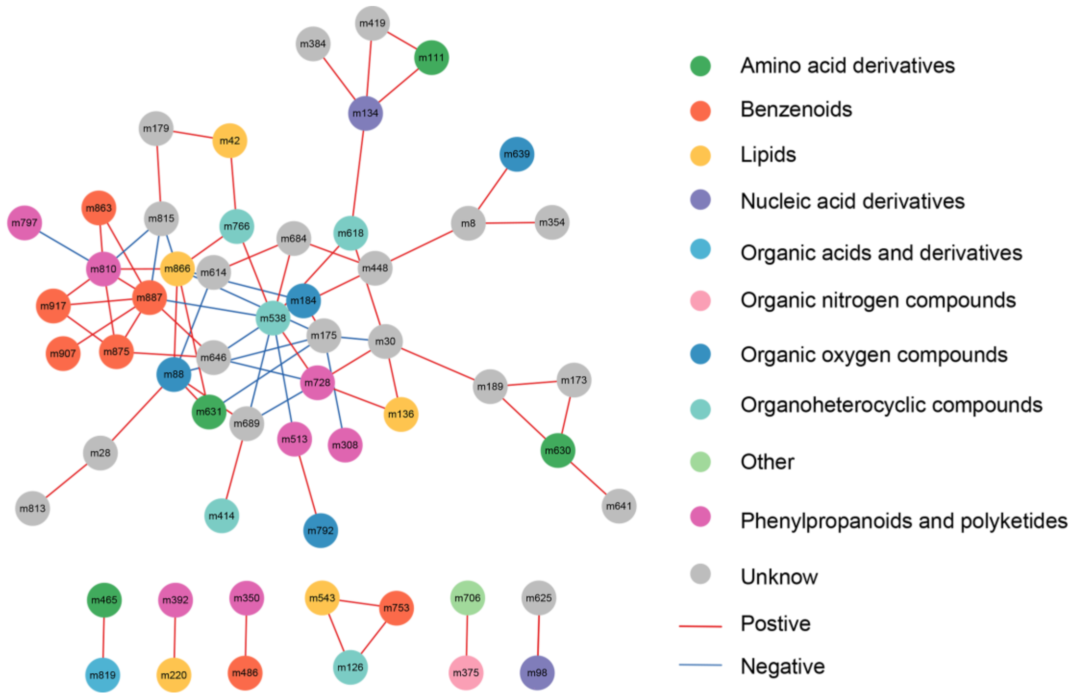

Supplemental Figure 5. Network visualization of 78 metabolic markers in maize. Metabolic markers are represented as nodes, while their correlation coefficient values are depicted as edges. The absolute values of Spearman's rank correlation coefficients above the threshold (r = 0.3) are displayed. Red indicates a positive correlation, while blue indicates a negative correlation. Metabolites from different chemical groups are marked by distinct colors, as shown on the right.

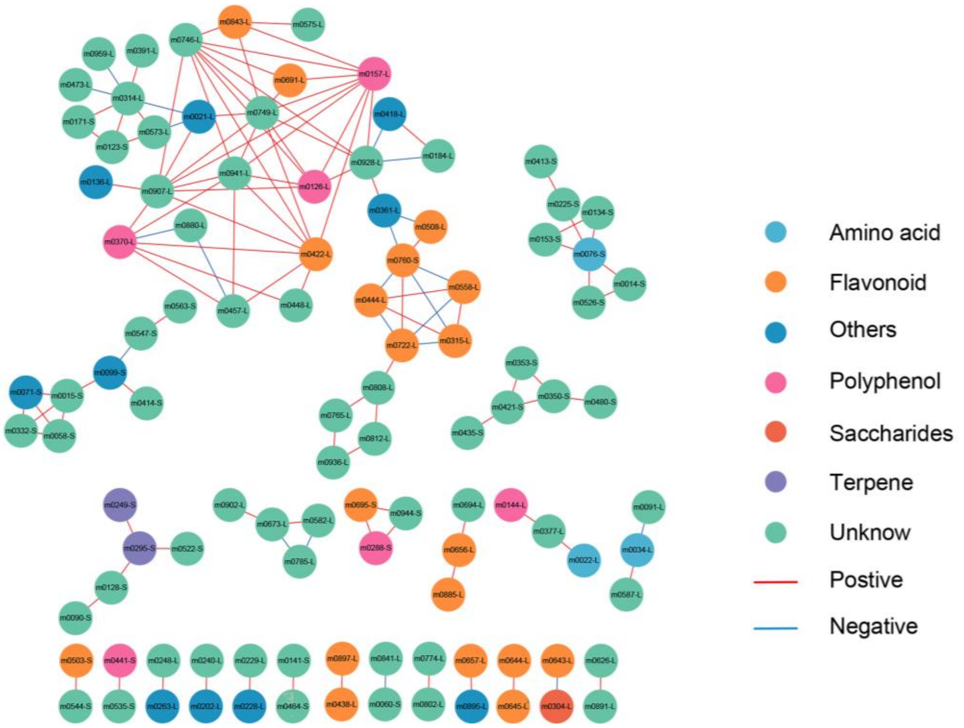

Supplemental Figure 6. Network visualization of 171 metabolic markers in rice. Metabolic markers are represented as nodes, while their correlation coefficient values are depicted as edges. The absolute values of Spearman's rank correlation coefficients above the threshold (r = 0.5) are displayed. Red indicates a positive correlation, while blue indicates a negative correlation. Metabolites from different chemical groups are marked by distinct colors, as shown on the right.

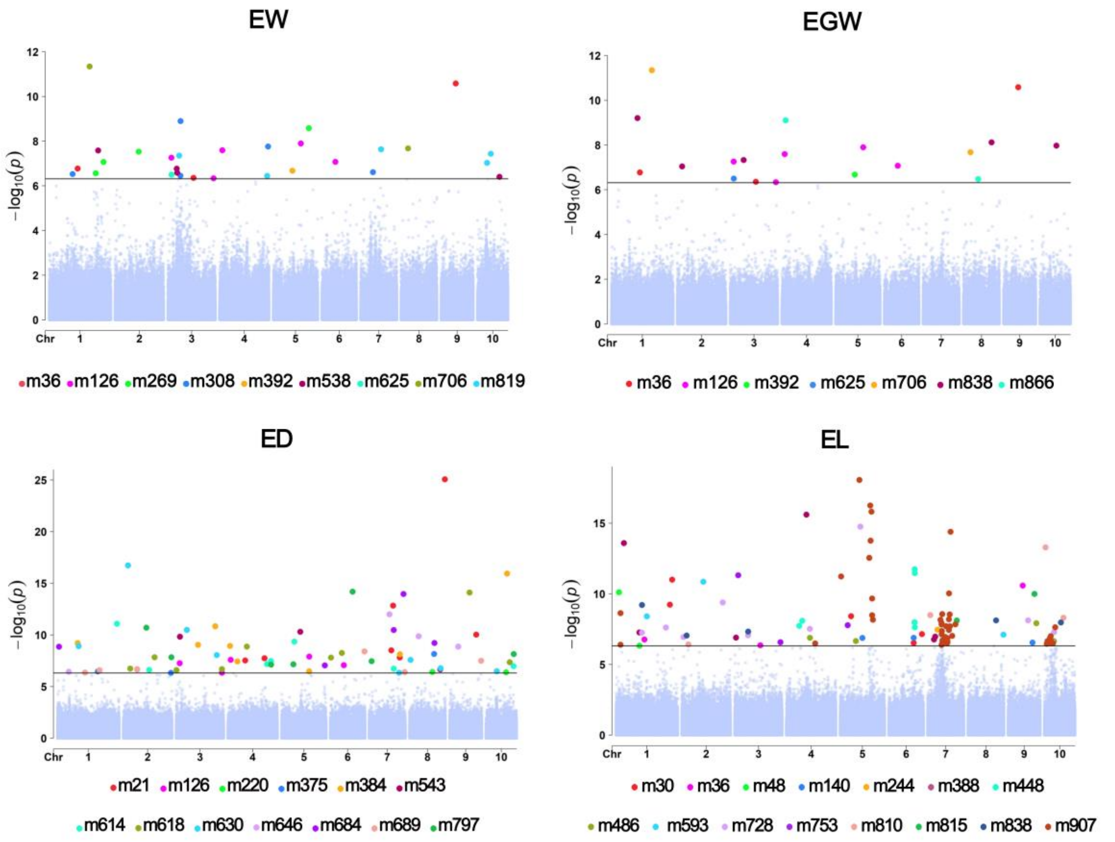

Supplemental Figure 7. Manhattan plots illustrating the results of mGWAS reveal genetic associations for metabolite markers in four maize traits. The strength of association for these metabolite markers is represented as the negative logarithm of the *P*-value obtained from the FarmCPU method. The horizontal black lines indicate the significance threshold of 0.05 for the Bonferroni-corrected *P*-value. Significant SNPs associated with different metabolic markers are depicted using distinct colors.

Supplemental Tables

Supplemental Table 1. Detailed information on 777 metabolites from 205 maize inbred lines.
(Separate Excel File)

Supplemental Table 2. Identification of metabolites associated with four traits in 205 maize inbred lines using the LASSO method.
(Separate Excel File)

Supplemental Table 3. Metabolites significantly associated with multiple traits in maize.
(Separate Excel File)

Supplemental Table 4. Enrichment pathways of metabolic markers in maize.
(Separate Excel File)

Supplemental Table 5. Identification of metabolites associated with four traits in rice RILs using the LASSO method.
(Separate Excel File)

Supplemental Table 6. Metabolites significantly associated with multiple traits in rice.
(Separate Excel File)

Supplemental Table 7. Predicted EW for all 20,910 potential crosses derived from 205 maize inbred lines using MM_GP.
(Separate Excel File)

Supplemental Table 8. Predicted YIELD for all 21,945 potential crosses derived from 210 rice RILs MM_GP.

(Separate Excel File)

Supplemental Table 9. Spearman's rank correlation between the metabolic markers in maize.

(Separate Excel File)

Supplemental Table 10. Spearman's rank correlation between the metabolic markers in rice.

(Separate Excel File)

Supplemental Table 11. Phenotypic variation explained for 78 metabolic markers in maize by parental genotypes.

(Separate Excel File)

Supplemental Table 12. SNPs significantly associated with metabolite markers in maize.

(Separate Excel File)

Supplemental Table 13. The gradient of the mobile phase in positive ion mode.

(Separate Excel File)

Supplemental Table 14. The gradient of the mobile phase in negative ion mode.

(Separate Excel File)
